# Natural transformation of *Thermotoga* sp. strain RQ7

**DOI:** 10.1186/1472-6750-14-39

**Published:** 2014-05-10

**Authors:** Dongmei Han, Hui Xu, Rutika Puranik, Zhaohui Xu

**Affiliations:** 1 Department of Biological Sciences, Bowling Green State University, 43403 Bowling Green, OH, USA

**Keywords:** *Thermotoga*, Natural transformation, Natural competence, pDH10, pDH12

## Abstract

**Background:**

*Thermotoga* species are organisms of enormous interest from a biotechnological as well as evolutionary point of view. Genetic modifications of *Thermotoga* spp. are often desired in order to fully release their multifarious potentials. Effective transformation of recombinant DNA into these bacteria constitutes a critical step of such efforts. This study aims to establish natural competency in *Thermotoga* spp. and to provide a convenient method to transform these organisms.

**Results:**

Foreign DNA was found to be relatively stable in the supernatant of a *Thermotoga* culture for up to 6 hours. Adding donor DNA to *T.* sp. strain RQ7 at its early exponential growth phase (OD_600_ 0.18 ~ 0.20) resulted in direct acquisition of the DNA by the cells. Both *T. neapolitana* chromosomal DNA and *Thermotoga-E. coli* shuttle vectors effectively transformed *T.* sp. strain RQ7, rendering the cells resistance to kanamycin. The *kan* gene carried by the shuttle vector pDH10 was detected by PCR from the plasmid extract of the transformants, and the amplicons were verified by restriction digestions. A procedure for natural transformation of *Thermotoga* spp. was established and optimized. With the optimized method, *T.* sp. strain RQ7 sustained a transformation frequency in the order of 10^-7^ with both genomic and plasmid DNA.

**Conclusions:**

*T.* sp. strain RQ7 cells are naturally transformable during their early exponential phase. They acquire DNA from both closely and distantly related species. Both chromosomal DNA and plasmid DNA serve as suitable substrates for transformation. Our findings lend a convenient technical tool for the genetic engineering of *Thermotoga* spp.

## Background

*Thermotoga* species are a group of Gram-negative, strictly anaerobic bacteria that grow up to 90°C. Ever since their discoveries in 1980s, they have been attracting great attention due to their extreme life style and the underlining implications in both basic research and biotechnological applications. Most noticeably, they serve as excellent candidates for the production of hydrogen gas, which may be directly used as a clean fuel [1,2]. Producing biohydrogen using fermentative hyperthermophiles like *Thermotoga* has many advantages, including independence from light sources, reduced risks of contamination, lower energy requirement for agitation and cooling, and above all, higher hydrogen partial pressure. To fully release the potentials of *Thermotoga*, one often wishes to modify these bacteria at the genetic level. However, *Thermotoga* spp. are considered genetically recalcitrant. Their first transformants were isolated only recently after decades of efforts [3]. Making these species more genetically accessible remains a priority for the research community. Effective delivery of recombinant DNA stands as a critical step in any genetic modification attempt dealing with *Thermotoga*. Previous studies have demonstrated that *Thermotoga* cells are transformable by liposome-mediated transformation and electroporation [3,4]. Effective as these methods are, they are labor-intensive and time-consuming. A more convenient approach would be highly desirable if one needs to transform *Thermotoga* on a daily basis.

Many prokaryotes possess the ability to take up DNA directly from the environment without human intervention, i.e. they are naturally competent. Some bacteria efficiently import DNA from any source, whereas others prefer to acquire homologous DNA through the recognition of specific uptake signal sequences [5,6]. The process of natural transformation involves the steps of DNA binding, translocation, and recombination. In most competent bacteria, the machinery responsible for DNA binding and translocation is related to Type IV pili (T4P) and Type II secretion systems (T2S) [7,8]. T4P are retractile appendages that contribute to twitching motility, biofilm formation, adhesion, and conjugation, in addition to natural transformation. A working model proposed for *Neisseria gonorrhoeae* suggests that exogenous DNA crosses the outer membrane through a pore made of the secretin protein PilQ and transverses the periplasmic space with the aid of the PilE complex and a high affinity DNA-binding protein ComE. One strand of the DNA is then degraded by a nuclease, and the other strand travels across the inner membrane through a channel comprised of the membrane protein ComA [9,10]. In Gram-positives, DNA is transported across the cell envelope in a similar manner, except that it does not need to overcome the outer membrane barrier. Once in the cytoplasm, the single-stranded DNA has to be protected by single strand DNA binding proteins to avoid degradation by host nucleases. Recent evidence suggests that DNA protection protein DprA conveys incoming ssDNA to RecA, the ubiquitous recombinase responsible for the initiation of homologous recombination [11-13]. Protein ComM influences transformation at a very late stage presumably by affecting recombination of the donor DNA into the chromosome [14].

More than 60 bacterial species have been documented as naturally transformable [15], which actually represents a minute fraction of all the bacterial species characterized thus far. Natural transformation is therefore considered a rare phenomenon. However, competence genes are wide-spread in bacterial species, even though many of which have not been reported to be naturally competent. Part of the reason is that natural competence usually develops only under certain physiological states that are inducible by environmental factors. Often these conditions are ill-defined and thus cannot be reproduced in laboratory settings. It is very likely that natural competence occurs much more frequently than we observe. For example, this mechanism was only recently noted in such well-studied organisms as *Vibrio cholerae*[16], *Bacillus cereus*[17], and *E. coli*[18]. Natural uptake of DNA has not been reported for any bacteria growing optimally at, or above, 80°C, although this ability has been documented for hyperthermophilic archaeon *Thermococcus kodakarensis*[19].

Analyses of the published *Thermotoga* genomes present evidence of frequent lateral gene transfer events between *Thermotoga* and other bacteria, archaea, and even eukaryotes [20-23]. The chimeric nature of *Thermotoga* genomes suggests that these bacteria have a remarkable efficiency for acquisition of foreign genes. Although natural competence has not been reported for *Thermotoga*, putative competence genes have long been noticed in their genomes, including those encoding T2S- and T4P-related proteins [20]. Moreover, mini-plasmids pRQ7, pMC24, and pRKU1 have been discovered in *T.* sp. strain RQ7 [24], *T. maritima*[25], and *T. petrophila* RKU-1 [26], respectively. Therefore, it is possible that *Thermotoga* spp. are capable of acquiring foreign DNA directly. Searching of the *T. maritima* genome sequence did not uncover any specific uptake signal sequences [6]. For this reason, if *Thermotoga* spp. were to acquire DNA naturally, they should do so indiscriminately. The aim of this study was to establish natural competency in *Thermotoga* spp. and to develop a natural transformation protocol to facilitate the genetic engineering of *Thermotoga*.

## Materials and methods

### Strains and cultivation conditions

The bacteria strains and vectors used in this study are summarized in Table 1. *E. coli* strains were cultivated in Luria-Bertani broth (1% tryptone, 0.5% NaCl, 0.5% yeast extract) at 37°C. Ampicillin was supplemented at 50 μg per ml when required. *Thermotoga* strains were cultivated at 77°C in SVO medium, which was named by us after the first author of the paper where the medium was first described [32]. Gelrite of 0.075% and 0.3% were included for preparation of soft and solid media, respectively. When needed, kanamycin was supplemented at 150 μg ml^-1^ for liquid and 250 μg ml^-1^ for soft and solid cultures. To make a *Thermotoga* plate, the liquid culture was mixed with hot SVO Gelrite medium and poured into petri dishes. The plates were incubated in an anaerobic jar for 48 h at 77°C, and single colonies were picked up, inoculated into selective SVO soft medium to grow for 24 h, and transferred into liquid selective media for further propagation and analyses. The techniques for effective handling of *Thermotoga* cultures under atmospheric conditions were detailed in our previous report [3]. Cell densities were measured as optical absorbance at 600 nm (OD_600_).

**Table 1:** Strains & vectors used in this study

**Strain or plasmid**	**Description**	**Reference**
*T. neapolitana* DSM 4359	Isolated from African continental solfataric springs	[27]
*T. maritima* MSB8	Isolated from geothermally heated sea floors in Italy and the Azores	[28]
*T.* sp. strain RQ7	Isolated from geothermally heated sea floors in Ribeira Quente, the Azores	[28]
*Caldicellulosiruptor saccharolyticus* DSM 8903	Isolated from a thermal pool of Taupo, New Zealand	[29]
*E. coli* DH5α	F^-^*endA1 hsdR17* (r_k_^-^, m_k_^+^) *supE44 thi-1 λ*^*-*^*recA1 gyrA96 relA1 deoR* Δ(*lacZYA-argF*)- U169 ϕ80d*lacZ*ΔM15	[30]
**Plasmids**		
pKT1	pUC-derived plasmid, containing a *kan* cassette for thermostable kanamycin selections	[31]
pRQ7	Cryptic miniplasmid from *T.* sp. strain RQ7	[24]; GenBank: KF798180
pDH10	pRQ7 cloned between the EcoRI and XbaI sites of pKT1; Ap^r^; Kan^r^	[3]; GenBank: JN813374
pDH12	pRQ7 along with its duplicated fragment of 209 bp were cloned between the EcoRI and XbaI sites of pKT1; Ap^r^, Kan^r^	This study; GenBank: KF798179

### Construction of the *Thermotoga-E. coli* shuttle vector pDH12

**Figure 1:**
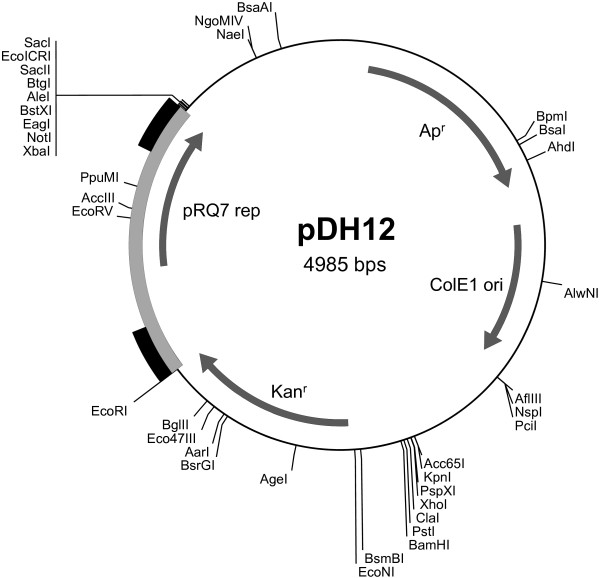
**The genetic and physical map of pDH12.** The bolded region in grey represents the sequence from pRQ7. The black bars denote the duplicated fragments. Unique enzyme sites are shown.

In the early stage of the study, pDH10, constructed in our previous study [3], was used as the substrate DNA to establish natural competency in *T.* sp. strain RQ7. In the late stage of the study, a new shuttle vector, pDH12 (Figure 1), was constructed. The two plasmids differ in two ways: 1) the *rep* gene and the *kan* gene are arranged in the same direction in pDH12 as opposed to a convergent orientation in pDH10; and 2) The upstream sequence of the *rep* gene is 416 bp in pDH12 and 207 bp in pDH10. The extra 209 bp fragment in pDH12, being included to enhance the expression of the *rep* gene, is a duplicate of the 3′-end of the *rep* gene.

To construct pDH12, a three-piece ligation strategy was adopted since the coding sequence of the pRQ7 Rep protein conveniently bears a HindIII site about a third way from its start codon. Primers 5′- GGGAATTCTGAAGTACTTATCAAAGGAG-3′ and 5′-CGTTTTTTAAGCTTTTCCCAGC-3′ were used to amplify the N-terminus of the *rep* gene and its upstream sequence, and the resulting amplicon carries a EcoRI site at its 5′-end. Primers 5′-GGGAAAAGCTTAAAAAACGAATCC-3′ and 5′-GCTCTAGATAATCACACTAACCACATTC-3′ were used to amplify the C-terminus of the gene; the corresponding amplicon carries an XbaI site at its 3′-end. The PCR program for both reactions included 30 cycles of 30 sec at 94°C, 30 sec at 54°C, and 1 min at 72°C. The PCR products were digested with EcoRI-HindIII and HindIII-XbaI, respectively, and were ligated to pKT1, pre-digested with EcoRI-XbaI, to give rise to pDH12. All DNA manipulations followed the standard methods.

### Natural transformation of *Thermotoga* spp

Two slightly different approaches were used. The early stage of the study involved the utilization of a nitrogen gas bottle. Briefly, 1 ml of an overnight culture of the recipient strain was transferred into 50 ml of fresh SVO medium and incubated to various growth stages. One ml of the fresh culture was then transferred to a 25 ml sterile serum bottle filled with N_2_ gas. At the same time, 50 μg of substrate DNA was added. After 6 h of incubation at 77°C with gentle agitation (100 to 125 rpm), the culture was mixed with hot SVO Gelrite medium and poured into petri dishes, with a supplement of 250 μg/ml kanamycin for selection. The plates were examined after 2 days of incubation at 77°C. Because the purpose of this study is to develop a simple method to transform *Thermotoga* spp., we intentionally did not apply DNase (to remove untransformed DNA after a designated period of time). This not only simplifies the overall procedure but also maximizes the occurrence of transformation events.

In the late stage of the study, the procedure was optimized as such: cells of 1 ml of the overnight culture of a recipient strain were collected by centrifugation, re-suspended in 200 μl of fresh SVO medium, and were injected into a 100-ml serum bottle containing 10 ml of fresh SVO medium; DNA substrate was added to a final concentration ranging from 5 to 10 μg/ml; the culture-DNA mixture was incubated at 77°C for 4 to 6 h with gentle agitation and then poured into selective plates (with a culture to medium ratio of 1 to 9, v/v). Transformation efficiency was computed as transformants per microgram of DNA, and transformation frequency was expressed as transformants per microgram of DNA per viable cell. The number of viable cells was determined by plating the control culture (no DNA added) with plain SVO solid medium. Plating efficiency was calculated as the ratio of viable cells to the total number of cells counted under microscope with Hausser Bright-Line 3120 Hemacytometer (Horsham, PA, USA).

## Results

### Stability of DNA in the transformation environment

**Figure 2:**
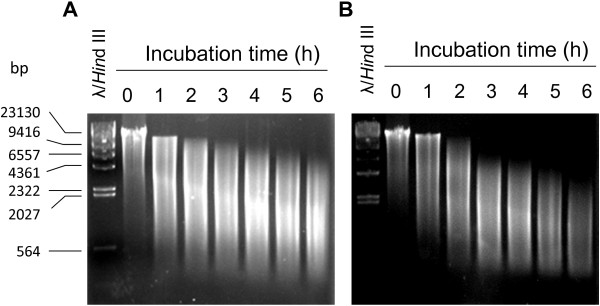
**Degradation of *C. saccharolyticus *** **genomic DNA in SVO medium (A) and in the supernatant of an overnight culture of *T. *** **sp. strain RQ7 (B).**

In natural transformation, naked DNA is directly exposed to the environment for many hours and is prone to degradation due to various physical, chemical, and biological forces, such as heat, pH, and nucleases. To maximize the chance of transformation, one would wish to incubate the DNA with the recipient cells for as long as possible. On the other hand, if the DNA has been degraded into small oligonucleotides, extended incubation will not lead to additional transformation events but instead cause the already transformed cells to age, which compromises plating efficiency and ultimately transformation frequency. Propagation of sibling transformants could be another concern. To find out how long heterologous DNA can survive under the growth conditions of *Thermotoga*, genomic DNA from *Caldicellulosiruptor saccharolyticus*, a Gram-positive bacterium with the optimal growth at 70°C, was incubated in plain SVO medium (pH 8.5) at 77°C for 6 h. Samples were withdrawn at hourly intervals and were analyzed with agarose gels (Figure 2A). Over the course of the experiment, the genomic DNA of *C. saccharolyticus* was graduately degraded into small fragments. By the end of the test the majority of the fragments were in the size range of 6.5 kb to 500 bp. Since the average length of a bacterial gene is ~ 1.1 kb, the size distribution of the *C. saccharolyticus* DNA fragments should cover most of the single genes as well as some operons, making the fragments an excellent pool of substrates for transformation.

Although DNA is fairly stable in pure SVO medium, it may not be so in a growing cell culture, where natural transformation is supposed to take place. The extracellular nucleases secreted by the host cells could expedite the degradation of foreign DNA. To take this biological factor into consideration, we next incubated the genomic DNA of *C. saccharolyticus* in the supernatant of an overnight culture of *T.* sp. strain RQ7, which contains high levels of free nucleases and is also more acidic (pH 5.5). Indeed, the foreign DNA experienced accelerated degeneration (Figure 2B). Within 3 h of incubation, the DNA fragments were around 6.5 kb or smaller, which was equivalent to 6 h of incubation in the pure growth medium. After 6 h, most DNA fragments were 4 kb or smaller. If DNA is added to a cell culture at an earlier growth stage (as was done in later experiments), its degradation profile is expected to fall between the two extreme scenarios shown here (Figure 2). Therefore, in a practical experiment, heterologous DNA may be incubated with *Thermotoga* cells for up to 6 h.

### Transformation of *T.* sp. strain RQ7 with *T. neapolitana* chromosomal DNA

**Figure 3:**
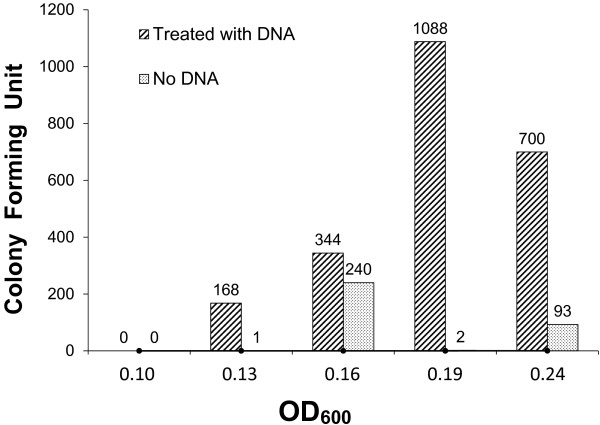
**Transformability of *T. *sp. strain RQ7 at various time points of the early exponential phase.** The number above each column represents the number of Kan^r^ colonies.

Among the three *Thermotoga* isolates available to us, *T. maritima*, *T. neapolitana*, and *T.* sp. strain RQ7, *T. neapolitana* is naturally resistant to kanamycin, whereas the other two are sensitive to it [3,4]. Accordingly, *T. neapolitana* served as the ideal donor strain and the other two the recipients. Transformation outcomes were measured by the acquisition of resistance to kanamycin (Kan^r^). In a series of preliminary tests (data not shown), we noticed that when *T. maritima* was used as the recipient strain, a few Kan^r^ colony were occasionally observed, despite when or whether the sample was treated with DNA. This indicates that these colonies were spontaneous mutants and, consequently, *T. maritima* was not naturally transformable under the tested conditions. The same was also true for the *T.* sp. strain RQ7 cells in their late log phase or stationary phase. Detailed experiments were then designed to focus on the early log phase of *T.* sp. strain RQ7 (OD_600_ = 0.1 ~ 0.25; Figure 3).

*T.* sp. strain RQ7 cells were tested at five time points: OD_600_ of 0.10, 0.13, 0.16, 0.19, and 0.24. The first time point did not yield any Kan^r^ colonies with either the treated sample or the control, probably because cells were not robust enough to be transferred to a N_2_ bottle, a process inevitably introduces trace amount of oxygen to the system. For the next four time points, the samples treated with the DNA generated significantly more Kan^r^ colonies than the controls did (Figure 3). The colony number of the treated samples peaked to 1088 if DNA was added at a cell density of ~ 0.19. If cells were allowed to grow for another 45 min and reached OD_600_ of 0.24, the number of Kan^r^ colonies dropped to 700, marking the decline of natural competence. The colony numbers from the controls varied from plate to plate, indicating that the mutation events happened randomly and independently in each N_2_ bottle rather than in the overnight seed culture or the fresh master culture from where samples were withdrawn.

**Figure 4:**
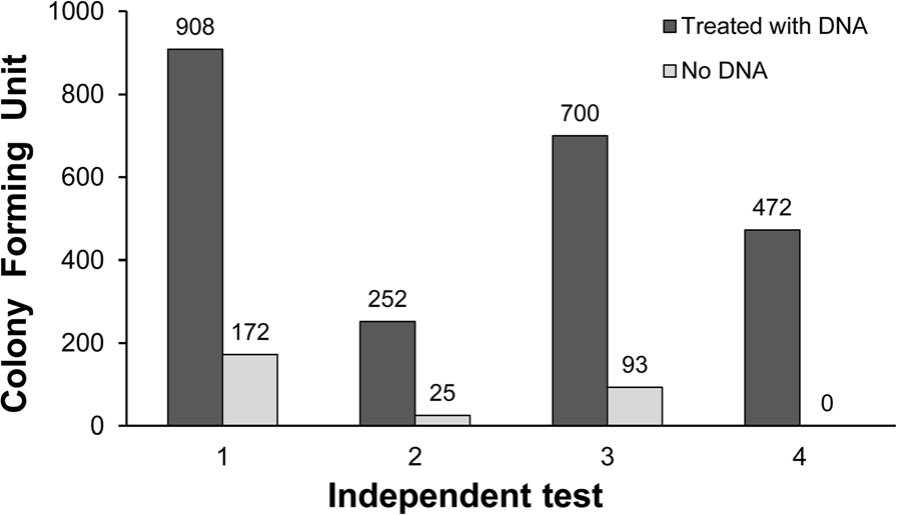
**Four more independent experiments of transforming *T. *sp. strain RQ7 at OD**_**600 **_**of 0.18 ~ 0.20.** The number above each column represents the number of Kan^r^ colonies.

The above results indicate that *T.* sp. strain RQ7 is most transformable when the cell densities are in the range of 0.18 - 0.20. We then repeated the experiment four more times under the same conditions (Figure 4). In these tests, the colony numbers fluctuated from time to time. This reflects a persisting challenge of handling *Thermotoga* cultures. Being strict anaerobes, *Thermotoga* cells are very sensitive to oxygen, especially during their active growth, which happens to be when they are mostly competent. A slight deviation in the handling time, especially in the process of transferring host cells to a N_2_ bottle, could greatly affect the viability of cells and hence the final results. Nevertheless, in these experiments, at least 5 times more kan^r^ colonies were obtained from the treated samples compared to the corresponding controls (Figure 4). Because the specific gene(s) responsible for kanamycin resistance in *T. neapolitana* are unknown, we were unable to validate the possible *T*. sp.RQ7 transformants at the molecular level. To address this deficiency and to demonstrate the application of natural competency in *Thermotoga*, we next transformed a shuttle vector to *T.* sp. strain RQ7.

### Transformation of *T.* sp. strain RQ7 with pDH10

Attempts were made to introduce the *Thermotoga*-*E. coli* shuttle vector pDH10 into *T.* sp. strain RQ7 and *T. maritima* through the aforementioned natural transformation procedure. The plasmid DNA was prepared from *E. coli* recombinant strain DH5α/pDH10. A total number of 132 colonies appeared on the transformant plates after 2 days, whereas the control plates had only 20 colonies arising from spontaneous mutations. No colony was observed with the *T. maritima* samples (including the controls), again indicating that this strain is not naturally transformable.

**Figure 5:**
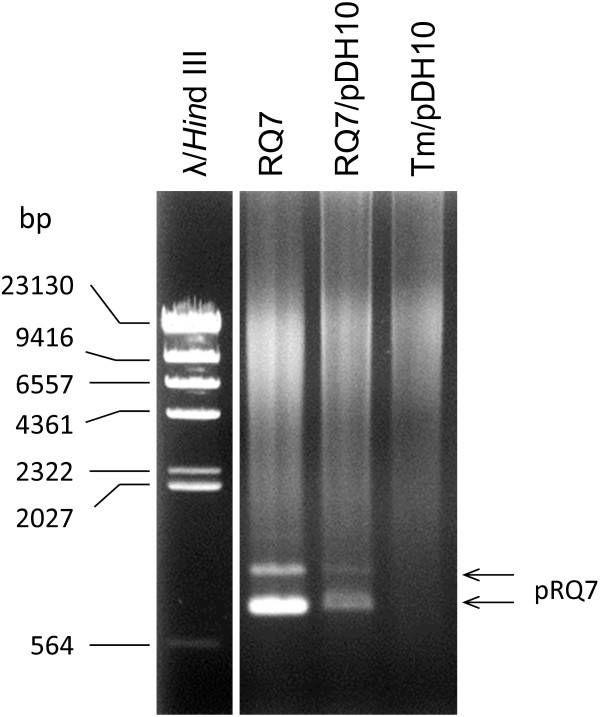
**Plasmid extract of a *T. *sp. strain RQ7/pDH10 transformant (denoted as RQ7/pDH10).** The host *T.* sp. strain RQ7 was used as the positive control (denoted as RQ7), and the recombinant strain *T. maritima*/pDH10, constructed in the previous study [3], served as the negative control (denoted as Tm/pDH10). The arrows point to the characteristic bands of pRQ7.

**Figure 6:**
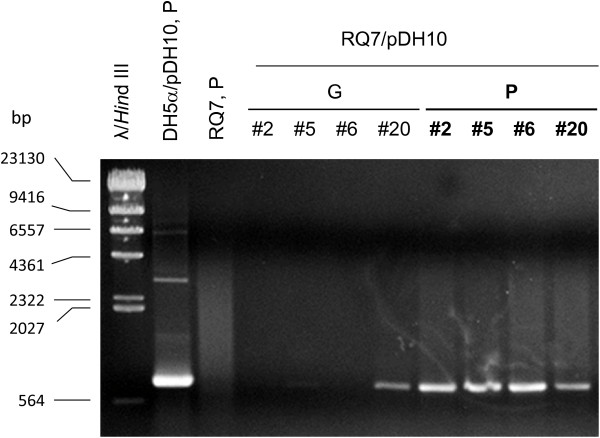
**Amplification of the *kan *gene from the genomic DNA (G) and the plasmid DNA (P) prepared from four RQ7/pDH10 transformants.** The plasmid extracts from DH5α/pDH10 (first lane after the marker) and *T.* sp. strain RQ7 (second lane after the marker) were used as positive and negative controls.

**Figure 7:**
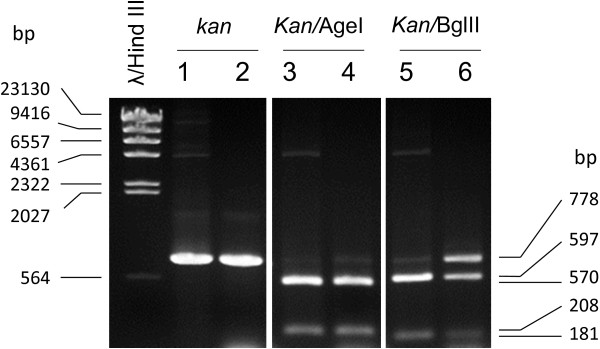
**Restriction digestions of the *kan *gene amplicon of a RQ7/pDH10 transformant.** Lanes 1, 3, and 5, positive controls prepared from DH5α/pDH10. Lanes 2, 4, and 6, RQ7/pDH10 transformant. Partial digestion is noticed in Lane 6.

Colonies of 20 potential RQ7/pDH10 transformants were picked for further analyses. Both plasmid and genomic DNA were extracted from four best growing cultures (#2, 5, 6, and 20). An example of the plasmid extract (#20) is shown in Figure 5. Same as before [3], bands corresponding to the size of pDH10 (4762 bp) were not observed; however, the characteristic bands of pRQ7 were clearly visible, indicating the plasmid extraction was successful. The plasmid extracts as well as the genomic DNA preparations were then subjected to PCR detection. Amplification using primers unique to the *kan* gene of pDH10 generated the expected band of 778 bp from all plasmid samples. By contrast, among the four genomic samples, only one had the band clearly visible (Figure 6). This implies that the transformed pDH10 survived as an autonomous replicon rather than an integrated part of the chromosome. To further confirm the identity of the amplicons, the PCR product was purified by ethanol precipitation and was digested by AgeI or BglII, which cut the *kan* gene into fragments of 208 + 570 bp and 181 + 597 bp, respectively. The amplicon indeed generated the expected sizes (Figure 7), confirming the authenticity of the PCR product. These data are in agreement with our previous study where the same vector was transformed into *T. maritima* and *T.* sp. strain RQ7 by electroporation and liposome-mediated transformation [3]. The previous data also demonstrated that pDH10 is stably maintained by the hosts [3].

### Optimization of natural transformation

We next devoted our efforts to simplifying the natural transformation procedure. The step of transferring host cultures into a N_2_ bottle was eliminated, because it was the major source of oxygen invasion. Instead, DNA was added at the time of setting up the fresh culture from the overnight seed. As a precaution of carrying over excessive nucleases from the overnight supernatant, cells were pelleted by centrifugation and were resuspended in fresh SVO medium prior to inoculation. To reduce the amount of DNA used, the total volume of the recipient culture was cut back from 50 ml to 10 ml and the final concentration of substrate DNA was decreased from 50 μg/ml to 5 μg/ml. Considering that cells may have a difficult time to bind DNA if the cell-DNA mixture was constantly shaken, we attempted to transform static *T.* sp. strain RQ7 cultures but did not get any transformants. Accordingly, the practice of gentle agitation was preserved.

The plating efficiency associated with cells under these physiological conditions were determined. Briefly, cells from overnight cultures were collected by centrifugation and were used to inoculate plain SVO, after 4 h of growth at 77°C, the culture was then subjected to microscopic cell counting (to determine the total number of cells) and was plated with plain SVO (to find out the number of viable cells). The results of three independent tests (Table 2) indicated that by the end of the transformation there were ~1 × 10^8^ viable cells per ml (CFU/ml), representing more than 70% of all cells found under the microscopy. The observed plating efficiency (73.4 ± 3.4%) was higher than what previously estimated (~50%) [3].

**Table 2:** **Plating efficiency of *T. *** **sp. strain RQ7 cells**

**Test**	**Viable cells (×10^8)**	**Total cells (×10^8)**	**Plating efficiency (%)**
1	1.20	1.70	70.7
2	0.97	1.25	77.2
3	0.97	1.34	72.3
Average	1.05	1.43	73.4
Standard deviation	0.13	0.24	3.40

Following the optimized procedure, we measured the transformation efficiency and frequency of *T.* sp. strain RQ7. The donor DNA was prepared from two sources: the chromosome of *T. neapolitana* and the plasmid pDH12, which is another *Thermotoga-E. coli* shuttle vector derived from pKT1 (Figure 1). *T.* sp. strain RQ7 presented a transformation frequency of (6.03 ± 1.52) × 10^-7^ (n = 3) with the genomic DNA and a frequency of (1.45 ± 0.02) × 10^-7^ (n = 2) with the plasmid DNA; for transformation efficiency, the figures were 16.6 ± 3.9 (n = 3) and 19.3 ± 3.8 (n = 2), respectively.

It was hoped that pDH12 would have a higher copy number than pDH10 because the extra sequence upstream of the *rep* gene may enhance the expression of this gene. However, pDH12 behaved in the same way as pDH10 did. The *kan* gene of pDH12 can be readily amplified by PCR and validated by restriction digestions, but attempts to identify the transformed plasmid by retransformation or inverse PCR have proven unsuccessful. A better understanding of the replication mechanism of pRQ7 will help to formulate new strategies on improving the copy numbers of pDH10 and pDH12.

## Discussion

### Transformability of *T.* sp. strain RQ7 and *T. maritima*

It is unclear why *T. maritima* is not naturally competent while *T.* sp. strain RQ7 is, even though the two strains are closely related. Because the genome sequence of *T.* sp. strain RQ7 is not publically available yet, we are unable to compare the putative competence genes across the two genomes. We speculate that the putative competency genes may be differentially regulated between the two strains, or some of the genes may have accumulated mutations leading to altered functionalities in one strain. It is also possible that there are novel natural competence genes that do not exhibit significant homology to known ones and therefore are beyond recognition by sequence comparison. More information should emerge once the genome sequence of *T.* sp. strain RQ7 is available. Studies on the expression of the putative competence genes should also yield important clues.

### Transformation frequency of *T.* sp. strain RQ7

With our optimized procedure, *T.* sp. strain RQ7 presented a transformation frequency in the order of 10^-7^. The chromosomal DNA and the plasmid DNA transformed this strain with comparable frequency, even though the plasmid DNA contained a higher mass ratio of the relevant gene(s), *i.e.* kanamycin-resistance gene(s). This is probably due to host restriction-modification systems, which selectively degrade DNA of distantly related species. A Type II RM system has been characterized in *T. neapolitana* and has also been found in *T. maritima*, *T. petrophila*, *T.* sp. strain RQ2, and *T. naphthophila*[33]. In addition, REBASE has identified a few putative methylases that might be involved in restriction-modification mechanisms in *Thermotoga*, such as M.TspRQ2ORF1808P in *T.* sp. strain RQ2 as well as M.TneDDamP and M.TneDORF1590P in *T. neapolitana* (http://rebase.neb.com). Because restriction nucleases have a low level of sequence conservation in general, it is much harder to predict their presence through bioinformatics analyses than finding methylases. Plasmid incompatibility is unlikely to be the primary reason for the lower than expected transformation frequency associated with pDH12, because plasmids containing the same origin of replication (*ori*) are more compatible than we previously thought. For instance, two plasmids carrying the same colE1 *ori* yet different antibiotic markers - kanamycin versus ampicillin - were found to be 100% compatible after five consecutive transfers under just kanamycin selection [34].

The level of competency observed in *T.* sp. strain RQ7 is comparable to what reported for *Ralstonia solanacearum*, whose frequency is in the order of 10^-7^[35], but lower than those measured in well-characterized system such as *Vibrio cholera*[36] and *Helicobacter pylori*[37], where a frequency of 10^-5^ or higher can often be obtained. It did not escape our attention that none of the putative competence genes found in *Thermotoga* are clustered. In many model systems, competence genes are often co-transcribed, such as the *com* operons in *Bacillus* and *Streptococcus*[15,38] and the *pil* operons in *Haemophilus* and *Thermus*[39]. The lack of the similar operons in *Thermotoga* suggests that their relevant molecular machinery either is not well-established or, to a lesser extent, represent a different type of machinery that does not share much similarity to those previously discovered. Nevertheless, the level of competence observed in *T.* sp. strain RQ7 sufficiently satisfies our engineering needs. As a matter of fact, we have been introducing recombinant DNA into *T.* sp. strain RQ7 exclusively by natural transformation ever since we discovered this mechanism.

## Conclusions

Natural competency has been established in *T.* sp. strain RQ7. A natural transformation procedure has been developed and optimized for *Thermotoga* spp.. This study provides an alternative way to transform these genetically recalcitrant yet biotechnologically attractive organisms.

## Abbreviations

Ap: Ampicillin; bp: Base pairs; CFU: Colony forming unit; DNA: Deoxyribonucleic acid; Kan: Kanamycin; ori: Origin of replication; PCR: Polymerase chain reaction; RQ7: *Thermotoga* sp. RQ7; Tm: *Thermotoga maritima*; Tn: *Thermotoga neapolitana*.

## Competing interests

The authors declare that they have no competing interests.

## Authors’ contributions

ZX conceived and coordinated the study and drafted the manuscript. DH discovered natural competence in *T.* sp. strain RQ7 and developed the initial transformation protocol. HX carried out the rest experiments. RP independently validated the results of transforming *T.* sp. strain RQ7 with pDH10. All authors participated in experimental designs and data analyses. All authors read and approved the final manuscript.
